# Neural Circuits on a Chip

**DOI:** 10.3390/mi7090157

**Published:** 2016-09-05

**Authors:** Md. Fayad Hasan, Yevgeny Berdichevsky

**Affiliations:** 1 Department of Electrical and Computer Engineering, Lehigh University, Bethlehem, PA 18015, USA; mdh415@lehigh.edu; 2 Bioengineering Program, Lehigh University, Bethlehem, PA 18015, USA

**Keywords:** neuron, culture, multiple electrode array (MEA), microstamping, optogenetic, microchannel, axon, circuit

## Abstract

Neural circuits are responsible for the brain’s ability to process and store information. Reductionist approaches to understanding the brain include isolation of individual neurons for detailed characterization. When maintained in vitro for several days or weeks, dissociated neurons self-assemble into randomly connected networks that produce synchronized activity and are capable of learning. This review focuses on efforts to control neuronal connectivity in vitro and construct living neural circuits of increasing complexity and precision. Microfabrication-based methods have been developed to guide network self-assembly, accomplishing control over in vitro circuit size and connectivity. The ability to control neural connectivity and synchronized activity led to the implementation of logic functions using living neurons. Techniques to construct and control three-dimensional circuits have also been established. Advances in multiple electrode arrays as well as genetically encoded, optical activity sensors and transducers enabled highly specific interfaces to circuits composed of thousands of neurons. Further advances in on-chip neural circuits may lead to better understanding of the brain.

## 1. Introduction

Neural circuits are responsible for the brain’s ability to process sensory information, recall memories, experience emotions, learn, and control the muscles of the body. Major approaches to the study of neural circuits include the examination of circuits in the intact brain in the context of relevant behavior as well as reductionist approaches where neural circuits or individual neurons are isolated from the rest of the brain for detailed electrophysiological and biochemical characterization. Isolation can be accomplished by dissecting a slice of the brain that contains a circuit of interest, or dissociating the brain into individual neurons and then allowing these neurons to form randomly connected networks in vitro. Advances in microfabrication and neuron culture techniques made it possible to control connectivity between dissociated neurons or slices. It is now possible to envision building living neural circuits of increasing complexity and precision to understand the brain’s ability to process and store information. Recent efforts toward this goal, including methods of circuit construction, stimulation, and detection of neural activity, and the achievement of pre-designed circuit functions are the subject of this review.

## 2. Dissociated Neural Cultures

**Figure 1:**
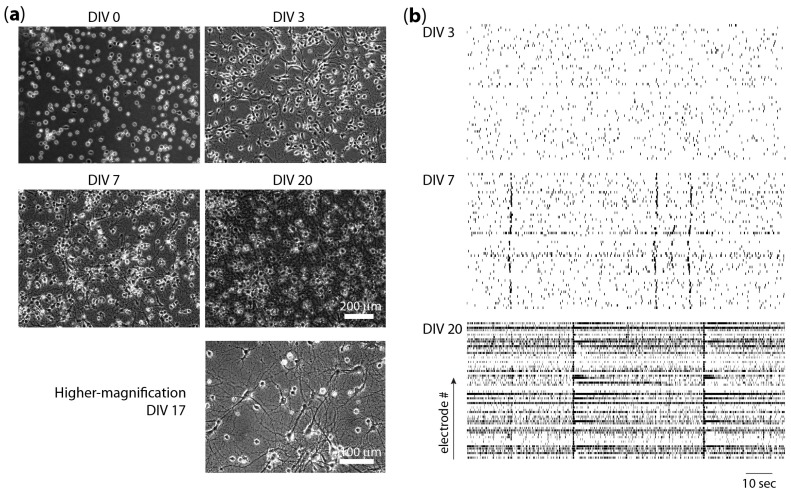
Randomly connected cultures (**a**) Phase micrographs of dissociated neuron cultures on day in vitro (DIV) 0, 3, 7, and 20 (scale bar = 200 µm), and a higher-magnification micrograph on DIV 17 (scale bar = 100 µm); (**b**) Spontaneous neural activity in dissociated neural cultures on DIV 3, 7, and 20. Raster plots were constructed from data published by Wagenaar et al., 2006 [6]. Each plot represents 2 min of activity recorded by 60 electrodes (vertical axis). Network-wide bursts of action potentials occurred on DIV 7 and 20.

Dissociated cultures of primary neurons are widely used in neuroscience [1,2]. The typical protocol starts with the dissociation of a particular brain region into individual cells and the plating of cells onto a substrate that has been coated to promote cell attachment. Culture medium that contains necessary nutrients and growth factors is added, and cultures are maintained at physiological pH and temperature. After plating, the neurons begin to extend processes that eventually differentiate into axons and dendrites (Figure 1a). Axon and dendrite growth is accompanied by the formation of synapses between cultured neurons [3,4]. Spontaneous activity in randomly connected cortical and hippocampal networks was analyzed by plating neurons onto planar multiple electrode arrays (MEAs). Recordings revealed that the frequency of spontaneous action potentials and synchronized bursts increased with time in culture (Figure 1b) [5,6,7]. Comparisons of low density and high density (number of neurons plated per area) cultures showed that the availability of postsynaptic partners influenced patterns of connectivity [8].

Cortical and hippocampal dissociated cultures are heterogeneous and include different neuron sub-types as well as glial cells. Most neurons in these cultures are excitatory and use glutamate as a neurotransmitter, but around 20% or more are inhibitory and release γ-Aminobutyric acid (GABA) [9,10,11,12]. Glial cells proliferate in culture medium formulations containing blood serum, but proliferation can be reduced by using mitotic inhibitors or chemically defined media [1,13,14].

Long-lasting alterations in synaptic strength in dissociated cultures can be induced by electrical stimulation [15,16]. These changes, termed synaptic plasticity, are thought to underlie certain forms of information storage in the brain [17]. Since studies of synaptic potentiation or depression were conducted via simultaneous intracellular recordings from neuron pairs or triples [18,19,20], researchers sought out a method to increase the probability that any given pair of neurons are connected. This was accomplished by developing protocols for very low density neural cultures [21], since the availability of postsynaptic partners influences the pattern of connectivity. Namely, neurons in sparse cultures were more likely to make a synapse with a nearby neuron than neurons in dense cultures [8]. More recent work relies on microtechnology to define neuron microislands to increase connectivity and is described in the Neural Patterning Methods section below. The availability of MEAs enabled simultaneous observations of activity of many neurons and led to the discovery of network-level plasticity in random dissociated cultures [22,23,24]. These studies examined synchronized bursts (Figure 1a) and showed that temporal and spatial activity patterns are stable and precise, and that long-term alterations can be induced by electrical stimulation. More recently, the presence of short-term information storage in dissociated random networks has also been demonstrated [25]. However, dissociated neuronal networks were limited to stimuli that did not evoke overly localized or largely overlapped responses in order to achieve successful learning [26].

## 3. Neural Culture Patterning Methods

**Figure 2:**
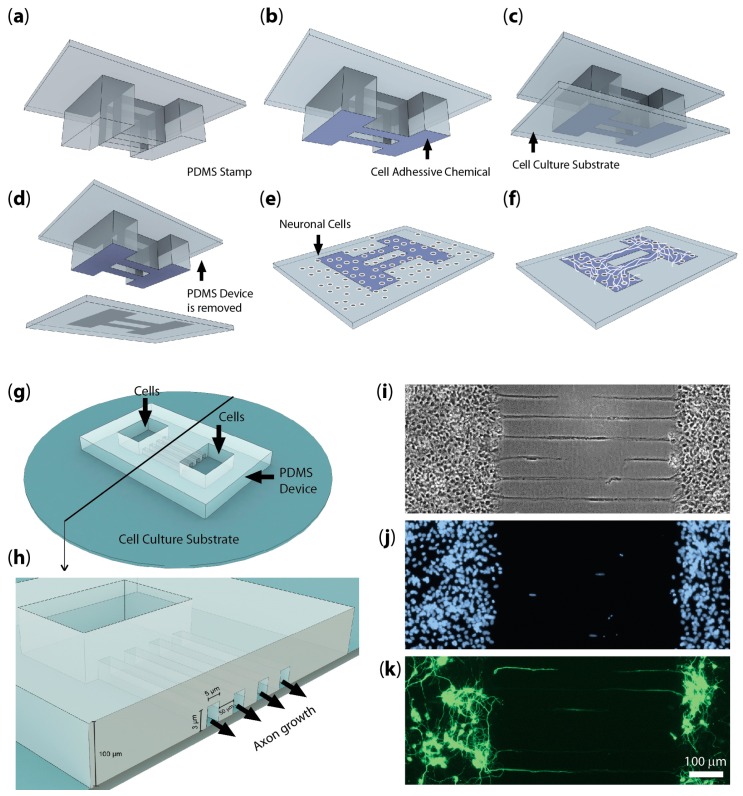
Techniques for patterning dissociated neuronal cultures: micro-contact printing with polydimethylsiloxane (PDMS) stamp (**a**–**f**); and physical confinement with a PDMS well/microchannel device (**g**–**k**). (**a**) Microfabricated PDMS stamp; (**b**) The bottom part of the device is submerged in a cell adhesive chemical; (**c**) A cell adhesive chemical attached to PDMS stamp is pressed against a cell culture substrate to print a cell adhesive pattern onto it; (**d**) The stamp is removed; (**e**) neurons are seeded onto the cell culture substrate; and (**f**) after adequate time needed for cell adhesion, the culture medium is changed. This removes the un-attached cells leaving the attached cells along the printed cell adhesive pattern. With adequately thin strip geometry, only axons can grow on thin portions of the pattern; (**g**) PDMS device containing wells for neuron cell bodies and microchannels for axon growth between wells; (**h**) Cross-section of PDMS device with dimensions (not drawn to scale), micro-channel openings are visible; (**i**) Bright field image of separated neuronal culture with axonal connection after removing PDMS device; (**j**) DAPI-stained nuclei and (**k**) anti-βIII Tubulin stained neuron soma, dendrites, and axons.

Dissociated neural cultures are composed of randomly connected neurons, unlike the precisely connected intact brain. Several techniques to control the connectivity in cultures were developed in order to study the relationships between circuit architecture and function, and to build more realistic neural circuits in vitro. Two of the most widely used techniques are based on: (1), controlling neuron location on a 2D substrate by creating patterns of cell-adhesive molecules (Figure 2a–f); and (2), creating 3D substrates that physically confine neurons to pre-designed locations (Figure 2g–k).

Cell adhesive molecules can be patterned by using silane and other types of chemistries on a photolithographically defined surface (for an early example, see [27]). The advent of soft lithography [28,29] made it possible to use photolithography to define stamps for microcontact printing of different molecules on glass and other substrates [30]. Polydimethylsiloxane (PDMS) stamps were used to create micro-regions of molecules that supported cell attachment and controlled cell location and shape [31]. This technique gained in popularity with the neuron patterning community since photolithography needed to be used only to create the stamp, which could then be used to pattern multiple substrates. The patterns of primary hippocampal neurons were created by defining regions of polylysine, a molecule that promotes neural attachment, by microstamping, and then coating the remaining area with polyethylene glycol (PEG) to prevent non-specific attachment [32]. Interestingly, patterned and random cultures had different levels of activity [33] and responded differently to stimuli [34]. It is also likely that interactions between neurons and glial cells such as astrocytes and oligondendrocytes are important for neural function. Astrocyte processes were found to guide neurites when neurons were patterned on astrocytes [35].

Physical neural confinement was accomplished in “neurochips” by building micromachined cages over the electrodes of a planar MEA [36]. The goal of this work was to permanently associate individual electrodes with individual neural cell bodies, and to have electrical access to all neurons in a small random network. This device required sophisticated microfabrication and did not gain wide-spread popularity [37]. A more accessible “NeuroArray” that used disposable PDMS stencils to confine single neurons or small groups was recently reported [38]; however, neural activity was detected with Ca^2+^ imaging rather than MEA. The PDMS stencil permitted confined neurons to connect randomly, similar to the “neurochip”.

The physical confinement method to control axon growth as well as neural cell body location was developed by Taylor et al. [39]. This technique relied on PDMS soft lithography to create wells for neural soma and microchannels for axons (Figure 2g–k). Microchannels were designed with a height of 3 µm, sufficient to allow axon growth cones to pass through, but too low for neural soma. This method was originally used to isolate axons for biochemical analysis, but it easily lends itself to studies of connected neural populations (discussed below). Since devices were fabricated by molding PDMS, they were easy to make, did not require sophisticated microfabrication facilities, and perhaps most importantly, were disposable, explaining the growing popularity of this method.

## 4. Patterned Networks

Perhaps the simplest application of neuron culture patterning to neuroscience was to create isolated networks of various sizes to study relationships between network size and connectivity [40]. PDMS stamps were used to define square cell-adhesive islands of laminin on a substrate onto which dissociated hippocampal neurons were then plated. Even these simple patterns led to interesting findings about the rules that govern neural network formation: when presented with more potential partners in larger networks, neurons traded off a few strong connections for more, weaker ones, while maintaining the same activity level regardless of network size.

In another study, photolithographically defined cell adhesion patterns were used to create cell body adhesion islands linked by adhesive lines for neurite (axon and dendrite) growth [9]. In close to 90% of cases, neurons on neighboring islands located 50 µm apart made monosynaptic (direct) excitatory and inhibitory connections, demonstrating the feasibility of creating neural circuits with defined connectivity. Neurons in culture extend multiple neurites, one of which differentiates into an axon while the rest become dendrites. Researchers hypothesized that the direction of axon growth and dendrite growth could be controlled by patterning the culture substrate. They created somatic adhesion sites with one solid adhesive strip and three punctuated strips for neurite extension. They found that a neurite growing from the soma along a solid adhesive strip preferentially differentiated into an axon, while neurites growing along punctuated strips became dendrites [41]. This method of controlling neuron polarity was later used to create oriented two-cell circuits of adult hippocampal neurons [42]. Neuronal polarization could also be controlled by micropatterned adhesion gradients [43] and by tuning the width and length of adhesion strips [44]. Circuits composed of neurite-extending individual neurons were also created using confinement devices [45,46,47].

A number of groups used physical confinement devices (Figure 2g–k) to create circuits composed of two or more axonally-linked neural populations [10,48,49,50,51,52,53]. Advantages of this approach include the ability to study two-stage feed-forward neural circuits [54] and to re-create in vitro neuronal pathways that are found in the brain. Some of the pathways re-created in vitro using multi-compartment devices include the cortico-hippocampal pathway [51], the mossy fiber pathway between DG and CA3 sub-regions of the hippocampus [10], and the thalamocortical [55] and cortico-striatial pathways [56]. A physical confinement array was used to enable a high-throughput screen of molecules that influence the formation of synapses, or synaptogenesis, in a neuron-fibroblast co-culture system [57]. Interestingly, electrical recordings from microchannel-confined axons revealed high signal-to-noise ratios due to high resistivity of microchannels [58,59].

**Figure 3:**
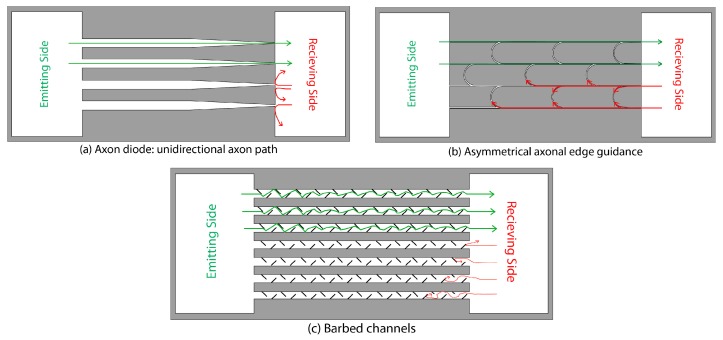
Different methods of forming uni-directional axonal path linking emitting neuronal population to receiving neuronal population in confinement devices. Green lines indicate allowed axonal growth and red lines indicate blocked axonal growth. (**a**) Channels with varying widths block most axonal growth from the narrow side and allow axonal growth from wide side. Adapted from [56] with permission of The Royal Society of Chemistry; (**b**) Asymmetric axonal edge guidance directs axons from the receiving end back through the bypass curved channels, effectively blocking their forward growth. Axons originating from the emitting side are not affected by bypass channels. Adapted from [61] with permission of The Royal Society of Chemistry; (**c**) Barbed channels deflect axons from the receiving side but allow axonal growth from the emitting side. Adapted from [49] under the terms of Creative Commons Attribution License (CC BY) https://creativecommons.org/licenses/by/4.0/, copyright © 2015 le Feber, Postma, de Weerd, Weusthof and Rutten.

Most pathways in the intact brain are asymmetrical; in other words, the strength of connection from region 1 to region 2 is often different than the reciprocal connection from region 2 to 1. In some cases (for example, pathways linking sub-regions of the hippocampal formation) the connections are unidirectional [60]. Several strategies were therefore developed to control directionality of the pathways re-formed in vitro. One strategy relied on plating neurons into corresponding wells at different times so that axons extended by one neuron population completely fill microchannels and prevent axons from another population from entering [50]. Other approaches were based on the inherent properties of axons: the “stiffness” that prevents growing axons from making sharp turns, and on the propensity of axon growth cones to follow walls and edges. The first approach utilized asymmetric microchannels that link two neuronal populations [56]. The goal was to have a strong connection from the emitting side to the receiving side, but not vice versa (Figure 3a).

Asymmetric channels were oriented such that the wide opening faces the emitting side, while the narrow opening faced the receiving side. More axons entered the wide opening compared to the narrow opening. Axons that successfully entered the microchannel followed the walls and emerged from the narrow opening. When only one side of the device was seeded with neurons, selectivity offered by this “axonal diode” approached 97%. More recently, a “return-to-sender” design was reported [61] where U-shaped channels return wall-following axons growing from the receiving side back to the other side. Axons growing from the emitting side are not affected by U channels because the angle of the turn is too sharp. Selectivity in this device depended on the number of U channels and reached 95% for three U-channels (Figure 3b), although it was lower when both neural soma compartments were seeded with neurons. Another recent approach relied on barbed channels, with barbs pointed in the desired direction of action potential propagation (Figure 3c) [49]. Control over axon directionality was also implemented in networks defined by the microprinting of cell-adhesion molecules. Daisy-chained populations of neurons with triangular geometry were created, and axon growth direction selectivity as well as directed neural activity propagation were achieved [62].

While progress in neural network patterning has been substantial, the current state of the art falls short of the precise positioning and connectivity of neurons in the brain. Technologies that are able to control a neuron’s fan-in (pre-synaptic partners) and fan-out (post-synaptic partners) at the individual synapse level have yet to be developed. Advances in micro- and nanotechnology and an improved understanding of neural development may need to be combined with the control of neural gene expression to form neural circuits that replicate those found in the brain.

## 5. Neural Circuits Carry Out Logic Functions

**Figure 4:**
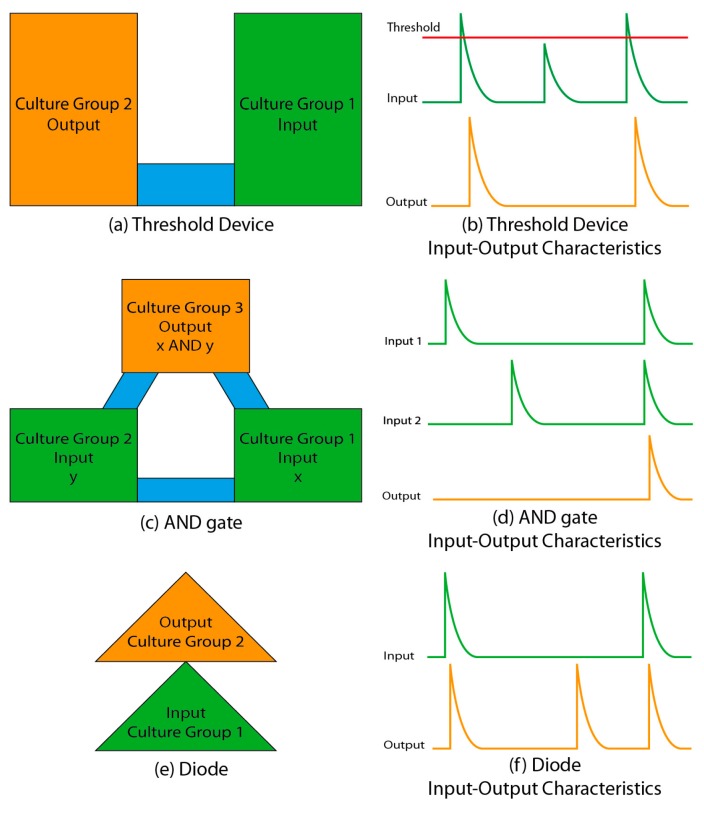
Neuronal implementation of functional logic gates. Adapted by permission from Macmillan Publishers Ltd.: [63] Left: Green color indicates input regions, blue shows bridges that contain axonal paths, and orange indicates an output region. Right: Green lines show conceptual input and orange lines show conceptual output (corresponding to fluorescence changes induced by changes in [Ca^2+^] during population bursts). The vertical axis corresponds to burst strength, while the horizontal axis represents time. (**a**) Threshold Device Geometry and its (**b**) Input-Output Characteristics; (**c**) AND gate, and its (**d**) Input-Output Characteristics; (**e**) Diode and its (**f**) Input-Output Characteristics.

Patterned dissociated neuronal cultures that carried out reliable logic operations were reported by Feinerman and colleagues [63]. These investigators used adhesive patterns of fibronectin/laminin to create neuronal patterns and utilized synchronized bursts that spontaneously occur in dissociated cultures (Figure 1) as units of activity. They constructed a threshold device by creating two neuronal populations linked by a thin cell-adhesive bridge (Figure 4a).

The thin bridge limited the number of axons linking two populations, and only the largest bursts (involving the most neurons) propagated from one population to another (Figure 4b). Two threshold devices connected in parallel formed a Boolean AND gate (Figure 4c), where the presence of a burst was a “1” and the absence of a burst was a “0”. Bursts had to occur simultaneously in the two input groups to successfully propagate to the output group. A diode-like device was constructed by chaining two triangular patterns together, which caused preferential axon growth from input group to output group (Figure 4e). This resulted in the burst propagation from input to output neural population, but not vice versa. Investigators also reported a neuronal oscillator and proposed designs for neuronal implementations of NAND and NOT logic functions.

Computation in these circuits required synchronized population activity and occurred on a hundreds of milliseconds to seconds timescale. Computation in the brain can be substantially faster and does not always require population bursts. Also, it is not clear that the brain’s neural circuits are organized into equivalents of Boolean circuits, or require individual circuit elements composed of neural clusters hundreds of micrometers wide. However, even with these limitations, work by Feinerman et al. is an important step in examining the capability of neurons to carry out logic operations. Future work in this area may focus on the experimental verification of theories of neural function and computation through the use of circuits composed of real biological neurons [64].

## 6. Structured Organotypic and 3D Dissociated Neural Cultures

Two dimensional cultures may not fully recapitulate the neuronal milieu of the intact brain; in particular, 2D cultures do not have the extracellular scaffolding that is required to sustain high cell densities and may lack extensive cell-to-cell interactions of the intact brain tissue [65]. Methods to produce 3D cultures were therefore developed to address the shortcomings of 2D cultures. These methods may be broadly divided into two categories: organotypic explant cultures, and dissociated neuron cultures organized into 3D constructs. Below, techniques to design neural circuits and re-create neural pathways using organotypic and 3D dissociated cultures are reviewed.

**Figure 5:**
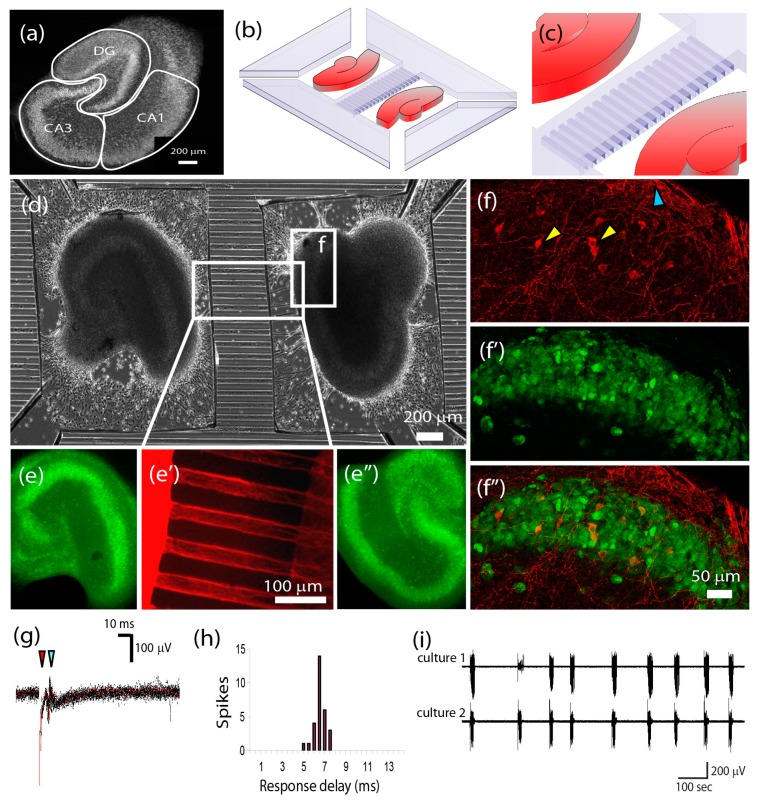
Pathway reconstruction using organotypic hippocampal cultures. (**a**) Single organotypic hippocampal culture. Layers containing neuronal soma appear bright in the image; (**b**) PDMS device to co-culture two organotypic hippocampal slices (slices colored red); (**c**) Microchannels connected to two PDMS wells; (**d**) Phase micrograph of the device; (**e**,**e”**) Cultures maintained well-organized neuronal layers, and (**e’**) axons grew from neurons in the cultures (**f**–**f”**), blue arrowhead points to axons that have been sprouted by neurons (indicated by yellow arrowheads); (**g**) Electrical stimulation of one hippocampal culture (red arrowhead) resulted in an evoked response in the neighboring culture (blue arrowhead) after a propagation and synaptic delay (**h**); (**i**) Spontaneous bursts in two axonally-linked cultures became synchronized after 14 DIV. (Reproduced by permission of The Royal Society of Chemistry [73]).

Organotypic cultures are created by dissecting a brain region of interest into slices of typically less than 500 µm thickness, and then maintaining those slices in physiological conditions in vitro for up to several weeks. Organotypic cultures become thinner with time in vitro, but even the thinnest cultures are composed of several stacked layers of neurons, unlike the 2D dissociated cell cultures. Wide applications of organotypic cultures have been reviewed by Gahwiler and colleagues [66]. The major advantage of organotypic over dissociated cultures is that the cytoarchitecture and some of the connectivity of the originating brain region are preserved (hence the term, “organotypic”), thus giving experimenters long-term, in vitro access to relatively realistic neural circuits. The disadvantages of organotypics include a requirement for specialized equipment to access single cells in the slice (including differential interference contrast or confocal microscopy and penetrating electrodes) and specialized culture techniques (such as roller drums or perforated culture substrates) [66,67]. These culture techniques complicate efforts to use organotypic cultures on chips, although special perforated or rocking MEAs have been developed for this purpose [68,69,70,71]. More recently, a method for maintaining organotypic cultures on planar, unperforated substrates has been reported [72]. This method was used to develop a multi-slice platform for drug discovery [73] and to re-create commissural and perforant pathways of the hippocampal formation [74]. The latter work (Figure 5) used a device similar to what has been reported for two-population dissociated cell circuits (Figure 2g–k), but created a functional axonal link between neuronal populations contained in organotypic cultures. 

Three-dimensional dissociated cultures may be generated by encapsulating dissociated neurons in a three-dimensional scaffold such as collagen or Matrigel [65,75]. These cultures remain three-dimensional for several weeks in vitro, although internal neuronal organization and synapse formation is random [75]. Thin collagen gels were patterned using photothermal etching to isolate single cells or groups of cells growing on top of the gel, or to control the direction of neurite elongation [76]. While this approach still used neural cell bodies that were arranged on a two-dimensional plane, investigators noted that neurite growth occurred in three dimensions. Three-dimensional neurite growth in collagen gel was controlled by AC electrokinetic forces to create non-intersecting neurite bridges [77]. Pautot and colleagues chose a non-gel based approach to create ordered 3D neuronal networks [78]. They allowed dissociated neurons to attach to silica beads forming a close-packed 2D hexagonal array. Bead size was chosen to be larger than that of a neuron in order to allow one or more neurons to attach to a single bead. Beads with attached neurons were then transported to a different well, and 3D close-packed hexagonal arrays were assembled layer by layer. Cell density reached 75,000 cells per mm^3^, nearly reaching the density found in the brain [79]. After three weeks in culture, neuronal processes grew between beads, and functional synapses formed between layers. This approach may enable the construction of 3D neural circuits containing different cell types in each layer. Electrophysiological characterization determined that spontaneous and evoked activity were significantly different between 3D bead-based cultures and 2D cultures [80]. The bead-based scaffolding approach was extended to include microchamber population confinement and microgroove-guided connections [81]. A nanotechnology based approach used the electrospinning of nanofibers to create a membrane with arrayed microwells [82]. Dorsal root ganglion explants were then seeded into the wells, and neurites grew along the aligned nanofibers between adjacent wells. Although the resulting network was two-dimensional, this approach may be used to construct multi-layered networks in the future. A building-blocks technique that did not rely on the addition of exogenous scaffolding was used to pattern high-density 3D networks at the millimeter scale, and to re-create a cortico-hippocampal pathway in vitro [83].

## 7. Interfaces to Neural Circuits

Currently available and rapidly improving techniques enable the design and implementation of neural circuits in vitro as described earlier. Once a neural circuit is implemented, researchers typically need to determine how seeded cells behave in response to external stimulation and how they interact with each other. To answer these questions, numerous methods to observe the cell activities in vitro and means to stimulate them have been developed.

### 7.1. Electrical Interfaces

Methods for intracellular recordings of neuron membrane potentials have been developed decades ago [84,85]. This was a hallmark invention in the field of neurophysiology. Using a sharp electrode maneuvered carefully with a micromanipulator, the cell membrane was penetrated and cytosol was physically contacted. This kind of recording provides excellent coupling with selective single cell stimulation, and generates reliable data even for subthreshold activities [86]. However, due to the bulky micromanipulator and mechanical and biophysical instability, this type of recording can be done on only a few neurons at a time, thereby limiting applications to very small circuits [18,19].

Several techniques have been developed to provide electrical access to larger neural circuits, including substrate-integrated multiple electrode arrays for extracellular recordings [87]. In these arrays, electrodes are printed on the surface and then cells are seeded on the substrate and grown into patterned or random circuits (see Figure 1b) for examples of data recorded by a substrate-integrated MEA from a randomly connected network [6]). Extracellular electrodes are capable of recording and distinguishing activity from multiple neurons [88,89], and thousands of electrodes can be integrated onto a single MEA chip [90]. Therefore, this technology is capable of detecting the activity of 10,000 or more individual neurons. A major advantage of extracellular electrical recording methods is the high time resolution that enables the detection of single action potentials even in synchronized bursts. Some disadvantages include the non-specificity of stimulation (all excitable cellular compartments in the vicinity of a current-passing electrode, including nearby neural soma as well as passing axons, may become stimulated) as well as the relatively high cost of the arrays. While some investigators re-use MEAs for multiple experiments, the cost of individual chips makes it difficult to obtain the high sample numbers required for statistical significance in neuroscience experiments. The inability to treat MEA chips as disposable also prevents some experiments, such as on-chip immunohistochemistry that may render the chip toxic [50].

### 7.2. Optical Interfaces

Calcium channels are ubiquitous in neuronal membranes, and their activity-dependent activation makes it possible to detect synaptic and action potentials by optically monitoring changes in [Ca^2+^] [91]. Calcium imaging can be accomplished by loading neurons with Ca^2+^—sensitive fluorescent dyes [92] or by expressing genetically encoded calcium indicators, and then monitoring changes in fluorescence in individual cells with a microscope [93]. Unfortunately, Ca^2+^ dynamics in neurons are relatively slow, making it difficult to distinguish individual action potentials in bursts [94]. Voltage-sensitive dyes [91] and genetically encoded voltage indicators have also been developed [95], but issues with their signal-to-noise ratios and kinetics have not been completely resolved [91,96] (but see [97] for a recent report of high-speed recordings with a genetically encoded voltage indicator). 

Another advancement in biomolecular technology is the use of retinylidene proteins, also known as rhodopsins. Channelrhodopsins are light-sensitive ion channels [98] that can be artificially expressed in neurons and can be used to control neural activity with pulses of light [99]. A major advantage of genetically encoded activity indicators and channelrhodopsins is that their expression in neurons can be driven by promoters that are specific to only certain classes of neurons. Since genetically encoded calcium indicators are available in multiple wavelengths [100,101], multiplexed systems that activate and detect the activity in defined neuron populations can be envisioned [102].

A major advantage of optical interfaces to in vitro neural circuits is that the expensive equipment (light sources, microscope) is not in intimate contact with the cultures, as is the case with MEAs. Patterned culture dishes, PDMS stencils, or colloids can be engineered to be transparent and disposable, making it easier to run experiments that require large sample numbers. Fast multiphoton scanning microscopy enables the imaging of activity in 3D circuits [75]. Disadvantages of optical interfaces include slow kinetics compared to sampling rates that can be achieved with electrodes, and the requirement for relatively high light intensity to activate channelrhodopsins. Patterned stimulation with light of large-area neural circuits can therefore be technically challenging [25].

## 8. Conclusions

Understanding the brain has been an endeavor that has occupied scientists for decades. While linking neurons and behavior currently requires in vivo studies, it is important to note that in vitro studies led to important mechanistic insights and discoveries, such as homeostatic and spike-timing-dependent plasticity [20,103,104]. These discoveries inform and shape our current understanding of learning, development, and memory. Advances in building and interfacing neural circuits on chips described in this review represent a major addition to neuroscience’s toolbox. We can anticipate that a full understanding of in vitro neuronal circuits, including mechanisms of data processing and storage and effect of stored information on the input-output relationship may be accomplished with further development of technologies reviewed here. A full description of how these small building blocks work may in turn provide the means to harness the abilities of the brain’s circuits to develop future generations of computational devices [64].

An understanding of neural circuits will likely require full access to all of the circuits’ inputs and outputs, complete control over their connectivity, and the ability to detect the activity of every neuron. The current state of the art allows this for circuits composed of only a few neurons, although controlling connectivity even in these small circuits has proved challenging. Much of the current mechanistic understanding of neuronal function has been gained from these tiny networks, while it has been estimated that even the mouse brain contains approximately 75 million neurons [105]. Accessing the entire brain seems like an insurmountable challenge at this point, but neural circuits described in this review may represent an intermediate and more tractable system. It is possible to envision that technological progress will grant full access to circuits composed of hundreds or thousands of neurons within the next decade, resulting in completely new insights into how the brain works.
